# Metastasiertes kastrationsresistentes Prostatakarzinom: Welche Sequenzen sind am sinnvollsten?

**DOI:** 10.1007/s00120-023-02212-3

**Published:** 2023-10-17

**Authors:** Jana Horak, Ulf Petrausch, Aurelius Omlin

**Affiliations:** 1 Zentrum für Urologie Zürich – Klinik Hirslanden, Witellikerstr. 40, 8032 Zürich, Schweiz; 2 Onkozentrum Zurich, University of Zurich and Tumorzentrum Hirslanden Zurich, Zürich, Schweiz; 3 Medical School, University of Nicosia, Nicosia, Zypern

**Keywords:** Androgendeprivation, Radioligandentherapie, PSMA-PET, Metastasen, Androgenrezeptor-Signalweg-Inhibitoren, Androgen deprivation therapy, Radioligand therapy, PSMA-PET, Metastatic prostate cancer, Androgen receptor pathway inhibitors (ARPI)

## Abstract

**Hintergrund:**

Beim fortgeschrittenen Prostatakarzinom bezeichnet man die Krankheitsprogression unter der laufenden Androgendeprivation (ADT) als Kastrationsresistenz (CRPC). Für die Behandlung stehen verschiedene therapeutische Modalitäten zu Verfügung (endokrine Therapien, Chemotherapien, PARP-Inhibition, Radionuklid- und Radioligandentherapie).

**Ziel der Arbeit:**

In dieser Arbeit werden praktische Aspekte und Überlegungen zur Sequenztherapie ausgeführt.

**Material und Methoden:**

Die Ausführungen beruhen auf den vorhandenen prospektiven Phase-III-Studien, welche einen klinisch relevanten und statistisch signifikanten Vorteil im radiographisch progressionsfreien und/oder Gesamtüberleben nachweisen konnten.

**Ergebnisse:**

Die Sequenztherapie ist neben vielen individuellen patientenbezogenen Faktoren abhängig von der Behandlung, welche Patienten in der hormonsensitiven Situation (mHSPC) erhalten haben. Nach Vorbehandlung mit ADT alleine oder ADT plus Docetaxel in der mHSPC-Situation, ist eine zusätzliche endokrine Therapie der Standard. Bei Progress unter einer kombinierten endokrinen Therapie, welche in der mHSPC-Situation gestartet wurde, ist aktuell Docetaxel für die Mehrheit der Patienten der Standard. Patienten mit Triplet-Vorbehandlung in der mHSPC-Situation können mit einer Radioligandentherapie oder mit einer Zweitlinienchemotherapie behandelt werden.

**Schlussfolgerung:**

Für Patienten mit mCRPC stehen verschiedene, aktive und gut verträgliche Therapiemöglichkeiten zu Verfügung. Der Einsatz richtet sich primär nach den bereits erfolgten Vortherapien aber viele weitere, individuelle Faktoren werden mitberücksichtigt.

## Abstract (en)

**Background:**

In advanced prostate cancer, disease progression during ongoing androgen deprivation therapy (ADT) is referred to as castration-resistant prostate cancer (CRPC). Various therapeutic modalities are available for its treatment, including endocrine therapy, chemotherapy, poly (ADP-ribose) polymerase [PARP] inhibition, radionuclide therapy, and radioligand therapy.

**Objectives:**

This review outlines practical aspects and considerations regarding treatment sequencing in mCRPC.

**Materials and methods:**

The findings are based on existing prospective phase 3 studies that have demonstrated clinically relevant and statistically significant benefits in radiographically progression-free and/or overall survival.

**Results:**

Sequential therapy, aside from numerous patient-specific factors, depends on the treatment patients received in the hormone-sensitive prostate cancer (mHSPC) setting. Following pretreatment with ADT alone or ADT plus docetaxel in the mHSPC context, additional endocrine therapy is the standard approach. In the event of progression under combined endocrine therapy initiated in the mHSPC setting, docetaxel currently serves as the standard for the majority of patients. Patients who received triplet therapy as a pretreatment in the mHSPC scenario can be treated with radioligand therapy or second-line chemotherapy.

**Conclusion:**

Various active and well-tolerated treatment options are available for patients with metastatic castration-resistant prostate cancer (mCRPC). The choice of therapy is primarily determined by previous treatments, but many other individual factors are also taken into consideration.

## Einleitung

Wenn sich bei Patienten, die an einem fortgeschrittenen Prostatakarzinom leiden und bereits einer Hormonentzugstherapie (ADT) erhalten haben, Anzeichen für einen weiteren Krankheitsprogress zeigen, wird dies als Kastrationsresistenz bezeichnet. Die Wahl der geeigneten Behandlung in dieser Situation hängt von mehreren Faktoren ab, darunter frühere Therapien, Ausmaß und Dynamik der Krankheitsausbreitung, Symptome der Krankheit und Lebensqualität, gleichzeitig bestehende Krankheiten und begleitende Medikamente sowie die persönlichen Vorlieben des betroffenen Patienten.

## Grundsätzliche Überlegungen zur Behandlung von Patienten in der kastrationsresistenten Situation

Ein bestätigt steigender PSA-Wert (prostataspezifisches Antigen) auf ≥ 1,0 ug/l bei supprimiertem Testosteron (≤ 50 ug/l bzw. ≤ 1,73 nmol/l) definiert die kastrationsresistente Situation (CRPC; [24]). Abhängig davon, ob in der konventionellen Bildgebung (Computertomographie und Skelettszintigraphie) Metastasen identifiziert werden können, wird ein nicht-metastasiertes (nmCRPC) und ein metastasiertes Stadium (mCRPC) formal unterschieden. In der folgenden Arbeit wird nur die metastasierte Situation behandelt.

Ein steigender PSA-Wert bei supprimiertem Testosteron definiert die kastrationsresistente Situation

Für die Entscheidung, welche Therapie infrage kommt, müssen eine Reihe von Faktoren erfasst werden, u. a. die Art und Dauer der vorherigen Therapie, die aktuelle Ausdehnung der Erkrankung (Bildgebung) und Krankheitsdynamik (z. B. PSA-Verdopplungszeit), krankheitsbedingte Symptome, der aktuelle Gesundheitszustand, allenfalls bestehende Grunderkrankungen und bereits etablierte medikamentöse Therapien und nicht zuletzt, v. a. wenn verschiedene Therapiemöglichkeiten zur Verfügung stehen, die Wünsche des individuellen Patienten. Bei Patienten ≥ 70–75 Jahre wird ein kurzes geriatrisches Screening empfohlen [6]. Patienten mit relevanten Einschränkungen im geriatrischen Screening bedürfen einer vertieften Beratung und u. U. einer angepassten Therapie.

Die folgenden Ausführungen gelten grundsätzlich für Patienten ohne relevante Einschränkungen. Die Ausführungen gelten auch grundsätzlich für Patienten, bei denen keine Entwicklung einer Prostatakarzinomvariante vermutet wird. Bislang gibt es keine einheitliche klinische, laborchemische oder pathologische Definition für die aggressive Prostatakarzinomvariante. Eine aggressive Variante wird vermutet, wenn eine ausschließliche oder sehr ausgedehnte viszerale Progredienz vorliegt bzw. wenn eine Krankheitsprogredienz auftritt bei stabilem oder sehr tiefem PSA-Wert oder bei osteolytischen Knochenmetastasen. Wenn möglich sollte mittels Biopsie ein Zweitkarzinom ausgeschlossen werden. An der Biopsie kann untersucht werden, ob eine entdifferenzierte Adenokarzinomvariante (häufig noch mit Nachweis von PSA und Androgenrezeptor [AR]) bzw. ob eine neuroendokrine Differenzierung (Expression von Synaptophysin, Chromogranin A, CD56) mit oder ohne PSA und AR-Expression oder gar eine rein kleinzellig-neuroendokrine Variante vorliegt. Das Biopsiematerial kann auch für eine weiterführende molekularpathologische Zusatzuntersuchung nützlich sein.

Entscheidend für die Therapiewahl in der kastrationsresistenten Situation ist die Vorbehandlung

Entscheidend für die Therapiewahl in der kastrationsresistenten Situation ist die Vorbehandlung, welche Patienten in der hormonsensitiven Situation (mHSPC) erhalten haben. Entsprechend werden in der Folge die verschiedenen Szenarien separat aufgeführt und diskutiert.

Beim Einsatz von allen Therapien ist das Monitorring der Erkrankung wichtig, denn inzwischen steht eine Vielzahl von therapeutischen Möglichkeiten zur Verfügung, so dass eine Krankheitsprogression in der Regel doch frühzeitig erfasst werden sollte. Mit einem Therapiemonitorring sollten auch Komplikationen frühzeitig erfasst werden können (z. B. eine drohende spinale Kompression) und nicht zuletzt stehen Kliniker und Klinikerinnen zunehmend in der Verantwortung beim Einsatz der modernen und in der Regel teuren tumorspezifischen Therapien.

Eine molekularpathologische Untersuchung am Tumorgewebe zur Identifikation von therapierelevanten Alterationen (v. a. *BRCA1/2*-Alterationen, andere HRR-Alterationen, defekte Mismatch-Reparaturproteine [dMMR] und Mikrosatelliteninstabilität) sollte in Patienten, die fit sind für eine zielgerichtete Therapie spätestens bei Progredienz unter einer neuen endokrinen Therapie (Abirateron, Apalutamid, Darolutamid, Enzalutamid, zusammengefasst ARPI) erfolgen.

Die Advanced Prostate Cancer Consensus Conference (APCCC), welche alle 2 Jahre klinisch relevante Fragen zur Behandlung von Patienten mit fortgeschrittenen Prostatakrebs diskutiert und über im Vorfeld erstellte Konsensusfragen abstimmt, hat sich 2022 spezifisch mit der Frage der Sequenztherapie beschäftigt, einige Resultate daraus sind in Tab. 1 zusammengefasst [15, 16]. In Tab. 2 sind die Resultate dargestellt für Patienten, welche nur kurz auf eine Therapie in der hormonsensitiven Situation angesprochen haben. Die Resultate unterscheiden sich doch deutlich in der Wahl der folgenden Therapie in der kastrationsresistenten Situation.

**Table 1:** 

Klinische Situation	Größte Zustimmung	Zweithöchste Zustimmung	Andere Optionen	Kommentar
*Keine HRR-Alteration*, *ADT alleine für mHSPC*	*93* *% ARPI*	4 % Docetaxel	3 % ARPI + PARP	*Konsens: ARPI*
*Keine HRR-Alteration*, *ADT* *+* *ARPI für mHSPC*	*83* *% Docetaxel*	9 % alternativer ARPI	4 % alternativer ARPI + PARP 4 % Radium-223	*Konsens: Docetaxel*
*Keine HRR-Alteration*, *ADT* *+* *Docetaxel für mHSPC*	*93* *% ARPI*	5 % ARPI plus PARP-Inhibitor	2 % Taxan	*Konsens: ARPI*
*Keine HRR-Alteration*, *ADT* *+* *Docetaxel* *+* *ARPI für mHSPC*	56 % ^177^Lutetium-PSMA	27 % Taxan-basierte Chemotherapie	9 % Radium-223 5 % alternativer ARPI 3 % alternativer ARPI + PARP	Kein Konsens

*ARPI* „androgen receptor pathway inhibitor“: Abirateron, Apalutamid, Darolutamid, Enzalutamid, *PARP* Polyadenylribosepolymerase, *ADT* Androgendeprivation, *PSMA* „prostate specific membrane antigen“, *mHSPC* metastasiertes hormonsensitives Prostatakarzinom, *HRR* „homologous recombination repair“

**Table 2:** 

Klinische Situation, Ansprechen ≤ 6 Monate in der mHSPC-Situation	Größte Zustimmung	Zweithöchste Zustimmung	Andere Optionen	Kommentar
*Keine HRR-Alteration,* *ADT alleine für mHSPC*	*54* *% Chemotherapie (Taxan oder platinbasierte Therapie)*	43 % ARPI	3 % ARPI + PARP	*Kein Konsens*
*Keine HRR-Alteration,* *ADT* *+* *ARPI für mHSPC*	*95* *% Chemotherapie (Taxan- oder platinbasierte Therapie)*	3 % alternativer ARPI	1 % alternativer ARPI + PARP 1 % Radium-223	*Konsens: Chemotherapie*
*Keine HRR-Alteration,* *ADT* *+* *Docetaxel für mHSPC*	*75* *% ARPI*	19 % Chemotherapie (Cabazitaxel oder platinbasierte Therapie)	5 % ARPI + PARP 1 % Radium-223	*Konsens: ARPI*
*Keine HRR-Alteration,* *ADT* *+* *Docetaxel* *+* *ARPI für mHSPC*	51 % ^177^Lutetium-PSMA	47 % Chemotherapie (Cabazitaxel oder platinbasierte Therapie)	1 % alternativer ARPI 1 % Radium-223	Kein Konsens

*ARPI* „androgen receptor pathway inhibitor“: Abirateron, Apalutamid, Darolutamid, Enzalutamid, *PARP* Polyadenylribosepolymerase, *ADT* Androgendeprivation, *PSMA* „prostate specific membrane antigen“, *mHSPC* metastasiertes hormonsensitives Prostatakarzinom, *HRR* „homologous recombination repair“

## ADT alleine für mHSPC

Für diese Situation, welche in Zukunft aufgrund der Kombinationstherapien in der hormonsensitiven Situation wahrscheinlich immer weniger häufig anzutreffen ist, gibt es sehr gute Evidenz aus 2 Phase-III-Studien für die endokrine Therapien mit Abirateron bzw. Enzalutamid [4, 21]. In den 2 Zulassungsstudien (PREVAIL und COU-302) wurden nur Patienten mit asymptomatische bzw. minimal symptomatischer Erkrankung eingeschlossen. Klassischerweise wurde für symptomatische Patienten eine Chemotherapie mit Docetaxel empfohlen. Angesichts der sehr guten Verträglichkeit der oralen Therapien spricht aber wenig gegen eine endokrine Therapie in der ersten Linie, vorausgesetzt es wird nicht eine aggressive Variante des Prostatakarzinoms vermutet und diese Patienten werden engmaschiger überwacht um eine Krankheitsprogression frühzeitig zu erfassen.

Patienten mit einer *BRCA*-Mutation profitieren von einer Kombination (ARPI- und PARP-Inhibition)

Die Daten der PROpel‑, MAGNITUDE- und TALAPRO-2-Studien haben den Vorteil einer früheren Kombinationstherapie (endokrine Therapie plus PARP-Inhibition [Polyadenylribosepolymerase]) in der ersten Linie der kastrationsresistenten Situation untersucht [1, 7, 8]. Präklinisch gibt es Evidenz für eine synergistische Wirkung der Kombinationstherapie, allerdings hat sich in den 3 Studien gezeigt, dass v. a. Patienten mit einer *BRCA*-Mutation von einer Kombination profitieren (radiographisch progressionsfreier Überlebensvorteil, rPFS). Der Effekt der Kombinationstherapie ist weniger ausgeprägt in Patienten mit anderen homologen Rekombinationsdefekten (HRR-Alterationen, „homologous recombination repair“) und noch etwas geringer im Patienten ohne Nachweis von HRR-Alterationen. Ein signifikanter Gesamtüberlebensvorteil konnte bis jetzt in keiner Studie nachgewiesen werden. Der Einsatz eines PARP-Inhibitors ist mit relevanter, v. a. hämatologischer Toxizität assoziiert und in allen 3 Studien gab es einen nicht unerheblichen Anteil an Patienten, welche die Therapie unterbrechen oder in der Dosis reduzieren mussten. Die Daten dieser Studien werden von den Zulassungsbehörden unterschiedlich interpretiert. Zur Zeit der Erstellung dieses Manuskripts gibt es eine Zulassung von der European Medicines Agency (EMA) für die Kombination aus Niraparib plus Abirateron für Patienten mit *BRCA*-Alteration und für Olaparib plus Abirateron für alle Patienten mit mCRPC, Talazoparib und Enzalutamid sind noch nicht in der Kombination zugelassen.

## ADT plus Docetaxel für mHSPC

Interessanterweise gibt es für diese Situation keine direkte Evidenz aus randomisierten Phase-III-Studien, da die neuen endokrinen Therapien entwickelt wurden, als Docetaxel noch nicht in der hormonsensitiven Situation als Standard zum Einsatz kam. Basierend auf 2 großen Phase-III-Studien (COU-301 und AFFIRM) gibt es aber gute Evidenz für die Wirksamkeit von Abirateron und Enzalutamid nach einer Vorbehandlung mit Docetaxel und entsprechend haben die Mehrheit der Experten an der Konsensuskonferenz für eine solche Option abgestimmt [10, 23].

Im klinischen Alltag muss die verbesserte Wirksamkeit gegenüber der Toxizität abgewogen werden

Grundsätzlich könnte auch in dieser Situation eine Kombination aus PARP-Inhibitor und endokriner Therapie zum Einsatz kommen, 20–30 % der eingeschlossenen Patienten hatten eine Chemotherapie vor Einschluss in die Studie erhalten. Es gelten die grundsätzlichen Überlegungen wie oben ausgeführt, im klinischen Alltag muss die verbesserte Wirksamkeit abgewogen werden gegenüber der zusätzlichen Toxizität, v. a. in Patienten, bei denen die molekularpathologische Untersuchung keinen Nachweis von HRR-Alterationen ergeben hat.

Die Abstimmungsresultate der Konsensuskonferenz zeigen, dass zum Zeitpunkt der Konferenz (April 2022) nur ein sehr geringer Anteil der Expertinnen und Experten eine Kombination aus endokriner Therapie und einem PARP-Inhibitor in Patienten ohne molekulare Alteration einsetzen würden.

Bei rascher Entwicklung einer Kastrationsresistenz nach Vorbehandlung mit Docetaxel in der hormonsensitiven Situation wäre auch direkt eine Zweitlinienchemotherapie mit Cabazitaxel eine denkbare Therapieoption. Für den Re-Challenge mit Docetaxel gibt nur limitierte Daten bzw. retrospektive Fallserien [2]. Die Aktivität von Docetaxel ist wie erwartet eingeschränkt und ein erneuter Einsatz sollte sorgfältig abgewogen werden, auch in Abhängigkeit vom Ansprechen beim ersten Einsatz und von ggf. bereits vorhandenen typischen Nebenwirkungen wie sensorischer Polyneuropathie.

## ADT plus endokrine Therapie (Abirateron, Enzalutamid oder Apalutamid: ARPI) für mHSPC

Die Mehrheit der Patienten, welche aktuell in der metastasierten, hormonsensitiven Situation eine systemische Therapie benötigen, werden mittels endokriner Kombinationen behandelt. Docetaxel ist aktuell die Standardfolgebehandlung für diese Patienten in der kastrationsresistenten Situation. Da sich unter einer endokrinen Therapie in der Regel ein langsamer PSA-Progress bei Entwicklung einer Kastrationsresistenz abzeichnet, besteht in ausgewählten Fällen bei bildgebend nachgewiesener Oligoprogression die Option einer lokalen Therapie um den Wechsel einer systemischen Therapie hinauszuzögern. Auch hierzu wurden 2022 an der Konsensuskonferenz Fragen diskutiert und im Manuskript ausgeführt [16].

Die Mehrheit der Patienten mit mHSPC werden mittels endokriner Kombinationen behandelt

Eine direkte Umstellung der endokrinen Therapie bei alleiniger PSA-Progression wird in der Regel nicht empfohlen. Basierend auf prospektiven Studien und aus den Daten der Patienten, welche im Kontrollarm von randomisierten Phase-III-Studien einen direkten Switch der endokrinen Therapie erhalten haben, zeigt sich ein klares und reproduzierbares Bild mit einer sehr geringen Antitumoraktivität der direkten sequenziellen endokrine Therapie [3, 12, 17, 22]. Einzig eine gewisse Wirksamkeit von Enzalutamid nach Abirateron ist nachgewiesen.

Für die Kombination aus ARPI und PARP-Inhibitor gibt es in dieser Situation kaum Evidenz, ein sehr geringer Anteil der Patienten in den 3 oben erwähnten Studien ist mit einem ARPI vorbehandelt worden. Allerdings gibt es gute Daten für die PARP-Inhibitor-Monotherapie (Olaparib, Rucaparib). Es besteht zzt. nur eine Zulassung für Olaparib und eingeschränkt auf Patienten mit pathogener *BRCA*-Alteration (Keimbahn und/oder somatisch; [9, 13]). Für Patienten mit anderen HRR-Alterationen ist in Europa zzt. kein PARP-Inhibitor zugelassen. Basierend auf der PROFOUND-Studie ist in den USA Olaparib auch für Patienten mit anderen DNA-Reparaturdefekten zugelassen. Die Daten, v. a. zu selteneren Alterationen sind noch gering. Für Patienten mit ATM-Alterationen ergibt sich aber aus mehreren Studien und Analysen zunehmend das Bild einer sehr eingeschränkten PARP-Aktivität [14].

Patienten mit symptomatischer Progredienz und Nachweis von metabolisch aktiven Knochen- und ohne viszerale Metastasen, die nicht fit sind für eine Chemotherapie mit Docetaxel können u. U. eine Radionuklidtherapie mit Radium-223 qualifizieren [20]. Die PSMAfore-Studie wiederum, welche die Radioligandentherapie mit ^177^Lu-PSMA-617 in chemotherapienaiven Patienten in einer großen, randomisierten Phase-III-Studie untersucht, verglichen mit einem Switch der endokrinen Therapie (siehe Ausführungen oben zur Aktivität eines solchen Kontrollarmes), hat gemäß Pressemitteilung den primären Endpunkt des rPFS erreicht, die Präsentation der gesamten Daten bleibt aber abzuwarten.

## ADT plus Docetaxel plus Abirateron oder ADT plus Docetaxel plus Darolutamid für mHSPC

Eine besondere Herausforderung in der Zukunft werden Patienten darstellen, welche in der hormonsensitiven Situation bereits eine 3fach-Kombinationstherapie erhalten haben (ADT plus ARPI plus Docetaxel). Für diese spezifische Patientenpopulation gibt es keine gute Evidenz aus prospektiven, randomisierten Phase-III-Studien. Einige der oben ausgeführten Überlegungen kommen auch in dieser Situation zum Einsatz. An der Konsensuskonferenz hat sich jeweils etwas mehr als die Hälfte der Expertinnen und Experten für den Einsatz der Radioligandentherapie mit ^177^Lu-PSMA ausgesprochen. Voraussetzung für den Einsatz dieser Therapie ist u. a. eine PSMA-PET-Bildgebung (Positronenemissionstomographie), welche eine relevante PSMA-Expression nachweist. Für Patienten mit *BRCA*-Alteration ist der Einsatz eines PARP-Inhibitors der Standard in dieser Situation (Tab. 1–3).

**Table 3:** 

Therapieoption	Vorbehandlung	Evidenz	Kommentar
Abirateron/Prednison	ADT alleine für mHSPC	COU-302 [21]	Gesamtüberlebensvorteil
Abirateron/Prednison	ADT alleine für mHSPC, Docetaxel für mCRPC	COU-301 [10]	Gesamtüberlebensvorteil
Enzalutamid	ADT alleine für mHSPC	PREVAIL [4]	Gesamtüberlebensvorteil
Enzalutamid	ADT alleine für mHSPC, Docetaxel für mCRPC	AFFIRM [23]	Gesamtüberlebensvorteil
Abirateron/Prednison + Olaparib	ADT, oder ADT plus Docetaxel (ca. 20 %) für mHSPC	PROpel [8]	rPFS-Vorteil am ausgeprägtesten in Patienten mit *BRCA*-Alteration, weniger in Patienten mit anderen HRR-Alterationen und am geringsten in Patienten ohne HRR-Alterationen
Abirateron/Prednison + Niraparib	ADT oder ADT plus Docetaxel (ca. 20 %), < 5 % ARPI für mHSPC oder nmCRPC	MAGNITUDE [7]	rPFS-Vorteil nur in molekular selektionierten Patienten, am ausgeprägtesten in Patienten mit *BRCA*-Alteration, weniger in Patienten mit anderen HRR-Alterationen
Enzalutamid + Talazoparib	ADT oder ADT plus Docetaxel (< 30 %), < 10 % Abirateron	TALAPRO‑2 [1]	rPFS-Vorteil am ausgeprägtesten in Patienten mit *BRCA*-Alteration, weniger in Patienten mit anderen HRR-Alterationen und am geringsten in Patienten ohne HRR-Alterationen
Olaparib	ADT, Enzalutamid oder Abirateron, Taxan (ca. 65 %)	PROFOUND [9]	OS-Vorteil in Kohorte A: *BRCA1/2-* und ATM-Alterationen Kein Vorteil (rPFS und OS) in Kohorte B: andere HRR-Alterationen
Rucaparib	ADT, Abirateron oder Enzalutamid	TRITON‑3 [13]	rPFS-Vorteil in Subgruppe von Patienten mit *BRCA1/2*-Alteration
Docetaxel	ADT alleine für mHSPC	TAX-327 [26] NCT00255606 [18]	OS-Vorteil Option 2‑wöchentlich Docetaxel
Cabazitaxel	ADT, Docetaxel (für mCRPC)	TROPIC [11] PROSELICA [5] FIRSTANA [19] CARD [12]	OS-Vorteil, OS-Vorteil gegenüber Abirateron oder Enzalutamid nach Docetaxel, kein OS-Vorteil für Cabazitaxel 25 mg/m^2^ gegenüber Cabazitaxel 20 mg/m^2^ (PROSELICA), kein OS-Vorteil für Cabazitaxel gegenüber Docetaxel als 1‑Linien-Chemotherapie (FIRSTANA)
Radium-223	ADT, Docetaxel (57 %) ADT alleine (43 %, nicht fit für Docetaxel)	ALSYMPCA [20] ERA-223 [25]	OS-Vorteil kein Vorteil für Kombination mit Abirateron/Prednison bzw. erhöhte Rate an Frakturen (ERA-223)
177Lu-PSMA	ADT, Docetaxel, ARPI	VISION [22]	OS-Vorteil

*ARPI* Abirateron, Apalutamid, Darolutamid, Enzalutamid, *PARP* Polyadenylribosepolymerase, *ADT* Androgendeprivation, *PSMA* „prostate specific membrane antigen“, *mHSPC* metastasiertes hormonsensitives Prostatakarzinom, *HRR* „homologous recombination repair“, *OS* „overall survival“, *rPFS* radiographisch progressionsfreier Überlebensvorteil

## Fazit für die Praxis

- Für Patienten mit metastasiertem kastrationsresistentem Prostatakarzinom (mCRPC) stehen mittlerweile verschiedene Behandlungsoptionen zur Verfügung. Insbesondere die endokrinen Therapien Abirateron und Enzalutamid (ARPI), die Kombination von Olaparib bzw. Niraparib mit Abirateron oder Talazoparib mit Enzalutamid, die Chemotherapeutika Docetaxel und Cabazitaxel, die Radionuklidtherapie Radium-223 sowie die Radioligandentherapie ^177^Lutetium-PSMA („prostate specific membrane antigen“). Bei älteren Patienten mit relevanten Komorbiditäten bzw. einer deutlichen Einschränkung im geriatrischen Assessment kann auch eine dosisadaptiere Therapie oder Watchful Waiting zum Einsatz kommen.
- Eine perfekte Therapiesequenz kann aktuell nicht definiert werden, viel mehr richtet sich die Sequenz der Behandlungen nach der individuellen Krankheitsausdehnung, vorhandenen Symptomen, Begleiterkrankungen und Medikamenten sowie bereits durchgeführten Therapien.
- Das Monitoring von Patienten mit mCRPC umfasst regelmäßige klinische Untersuchungen, Laboruntersuchungen und bildgebende Verfahren, die in der Häufigkeit je nach individuellem Risiko angepasst werden.
